# Dynamic and balanced regulation of the *thrABC* operon gene for efficient synthesis of L-threonine

**DOI:** 10.3389/fbioe.2023.1118948

**Published:** 2023-03-02

**Authors:** Ruxin Hao, Sumeng Wang, Xin Jin, Xiaoya Yang, Qingsheng Qi, Quanfeng Liang

**Affiliations:** State Key Laboratory of Microbial Technology, Shandong University, Jinan, China

**Keywords:** L-Threonine, *thrABC*, balance, dynamic, *ptsG*

## Abstract

L-threonine is an essential amino acid used widely in food, cosmetics, animal feed and medicine. The *thrABC* operon plays an important role in regulating the biosynthesis of L-theronine. In this work, we systematically analyzed the effects of separating *thrAB* and *thrC* in different proportions on strain growth and L-threonine production in *Escherichia coli* firstly. The results showed that higher expression of *thrC* than *thrAB* enhanced cell growth and L-threonine production; however, L-threonine production decreased when the *thrC* proportion was too high. The highest L-threonine production was achieved when the expression intensity ratio of *thrAB* to *thrC* was 3:5. Secondly, a stationary phase promoter was also used to dynamically regulate the expression of engineered *thrABC*. This strategy improved cell growth and shortened the fermentation period from 36 h to 24 h. Finally, the acetate metabolic overflow was reduced by deleting the *ptsG* gene, leading to a further increase in L-threonine production. With these efforts, the final strain P_*2.1*_-2901Δ*ptsG* reached 40.06 g/L at 60 h fermentation, which was 96.85% higher than the initial control strain TH and the highest reported titer in shake flasks. The maximum L-threonine yield and productivity was obtained in reported fed-batch fermentation, and L-threonine production is close to the highest titer (127.30 g/L). In this work, the expression ratio of genes in the *thrABC* operon in *E. coli* was studied systematically, which provided a new approach to improve L-threonine production and its downstream products.

## Introduction

**FIGURE 1:**
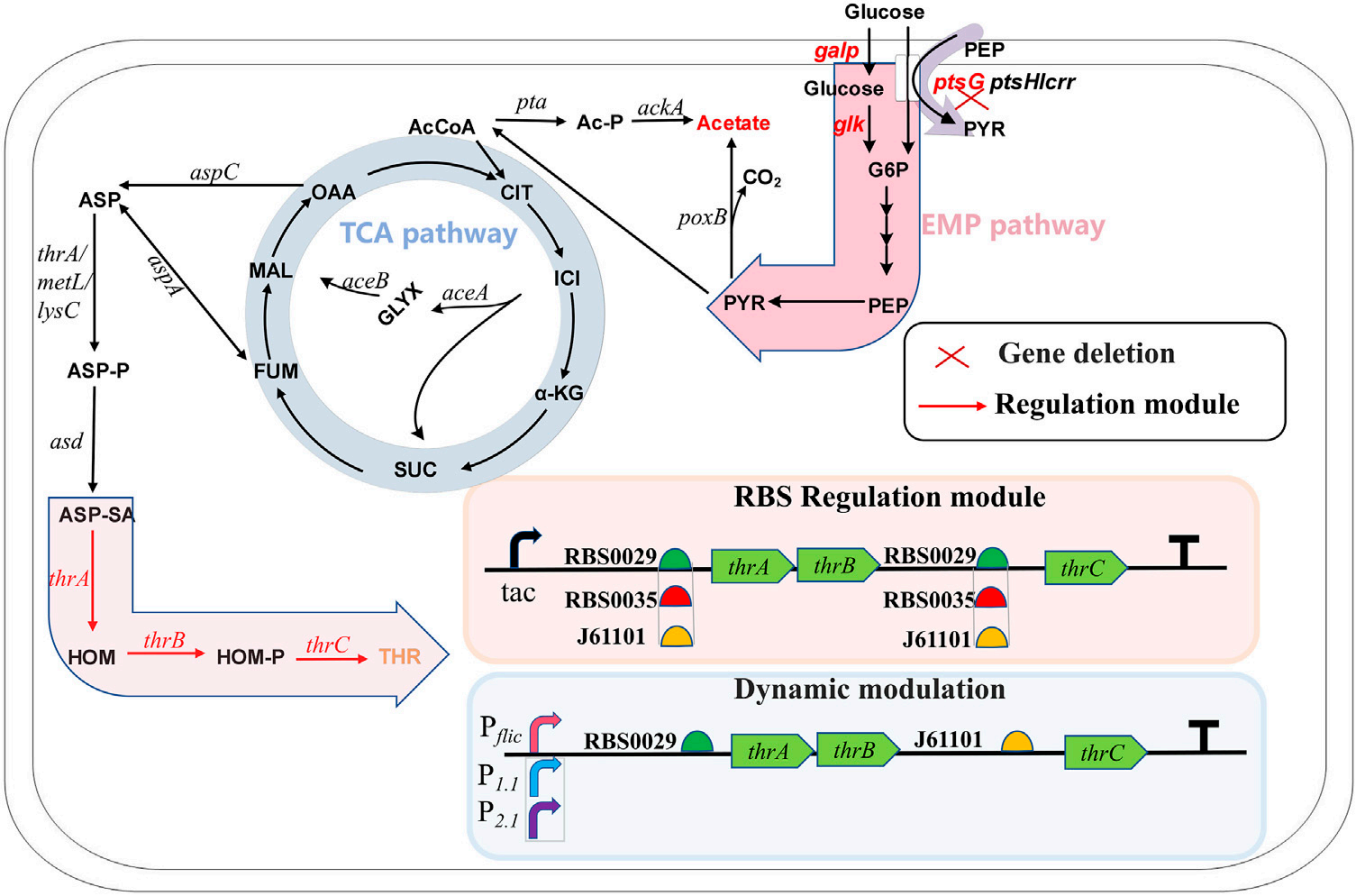
The metabolic pathway of L-threonine production in *Escherichia coli* and the modification strategy used in this study. G6P, glucose-6-phosphate; PEP, phosphoenol pyruvate; PYR, pyruvate; AcCoA, acetyl-coenzyme A; Ac-P, phosphorylated acetyl coenzyme A; CIT, citrate; ICI, isocitrate; GLYX, glyoxylate; α-KG, α-ketoglutarate; SUC, succinate; FUM, fumarate; MAL, malate; OAA, oxaloacetate; ASP, aspartate; ASP-P, aspartate phosphate; ASP-SA, aspartate semialdehyde; HOM, L-homoserine; HOM-P, homoserine phosphate; THR, L-threonine.

As the basic unit of proteins, amino acids play an important role in the nutrition and health maintenance of humans and animals (Lee et al., 2013). Among the 20 amino acids, L-threonine, as one of the nine essential amino acids, is used widely in food, cosmetics, animal feed and medicine. The market demand for L-threonine produced by microbial fermentation is increasing because of the growing demand for this amino acid (Wendisch, 2020). Amino acid synthesis starts at the glycolytic pathway when glucose is used as the carbon source. L-threonine biosynthesis uses the TCA cycle intermediate oxaloacetate or fumarate as a precursor. Aspartate transaminase, encoded by *aspC*, initially catalyzes oxaloacetate or aspartase, encoded by *aspA*, catalyzes fumarate to L-aspartate (Mu et al., 2021). Starting from L-aspartate, the L-threonine pathway consists of five enzymes that catalyze the reaction: aspartokinase encoded by *thrA*, *metL* or *lysC*; semialdehyde dehydrogenase encoded by *asd*; homoserine dehydrogenase encoded by *thrA*; homoserine kinase encoded by *thrB*; and threonine synthase encoded by *thrC* (Wang et al., 2022a) (Figure 1). Many strategies for synthesizing L-threonine have been explored, including overexpression of key genes, elimination of competing pathways and feedback inhibition, regulation of capacity and increased extracellular transport of L-threonine (Lee et al., 2007; Dong et al., 2011; Mu et al., 2021).

In *Escherichia coli*, *thrA*, *thrB* and *thrC* are arranged adjacently on the chromosome to form the *thrABC* operon. The *thrABC* operon controls several key enzymes, from L-Aspartate-4-semialdehyde to L-threonine synthesis. Research on *thrABC* expression and regulation is important for L-threonine synthesis and the downstream products, L-isoleucine and L-glycine. Overexpression of the *thrABC* operon in a bacterial strain increased L-threonine production significantly (Lee et al., 2003; Zhao et al., 2018), and an L-isoleucine-producing strain was constructed by overexpressing *thrABC* (Park et al., 2012). Threonine synthase encoded by *thrC* catalyzes the production of L-threonine from homoserine phosphate, which is the last step in the synthesis of L-threonine. Lee et al. reported that this reaction step is slower than reactions catalyzed upstream, and they studied how assembling homoserine dehydrogenase (HDH), homoserine kinase (HK) and different amounts of threonine synthase (TS) on the DNA scaffold affected the production rate of L-threonine (Lee et al., 2013). However, no study has examined the effect of reducing TS expression below that of HDH and HK. Recent studies have shown that regulating the ratio of different genes on the same operon is crucial for the efficient synthesis of products (Zelcbuch et al., 2013). Therefore, systematically studying the effect of varying the expression ratio of the *thrABC* operon genes may improve L-threonine production.

Dynamic regulation can prevent metabolic burden on cells caused by constitutive gene expression in L-threonine production. We previously designed a positive feedback strategy to dynamically regulate the expression of threonine transporters, which alleviated the adverse effects of overexpressed transporters on cells and excreted intracellular metabolites (Wang et al., 2022a). In addition, promoters of *cysH*, *cysJ* and *cysD* that can be activated by L-threonine control the expression of *aspC* gene, which did not affect cell growth and increased the production of L-threonine (Zhao et al., 2020).

In this study, we developed a new strategy for dynamic and balanced regulation of the *thrABC* operon for efficient synthesis of L-threonine (Figure 1). We first explored the optimal expression levels of *thrAB* and *thrC* to improve L-threonine production. We then used dynamic regulation to control the expression of *thrABC*. Finally, we deleted the PTS system in an effort to reduce metabolic overflow and further increase L-threonine production.

## Materials and methods

### Construction of strains and plasmids

The strains and plasmids used in this study are listed in Supplementary Tables S1, S2. *E. coli* DH5α was used for molecular cloning and manipulation of plasmids. All primers used are listed in Supplementary Table S3. All plasmids were constructed using the Gibson assembly method (Gibson et al., 2009). *E. coli* K-12 MG1655 was used for RBS strength characterization. RBS (B0029, B0030, B0031, B0032, B0033, B0034, B0035, B0064 and J61101) of different strengths were analyzed by the ratio of RFP to OD_600_. Nine plasmids were assembled. The expression of *thrAB* and *thrC* was regulated by using the primers listed in Supplementary Table S3, which replaced various RBS to construct L-threonine production plasmids (PA-29*thrAB-*29*thrC*, PA-29*thrAB-*01*thrC*, PA-29*thrAB-*35*thrC*, PA-01*thrAB-*29*thrC*, PA-01*thrAB-*01*thrC*, PA-01*thrAB-*35*thrC*, PA-35*thrAB-*29*thrC*, PA-35*thrAB-*01*thrC* and PA-35*thrAB-*35*thrC*). The TH strain producing L-threonine was provided by the Fufeng Group (Qingdao, China) and derived from *E. coli* K-12 MG1655. Three genes *thrA*, *thrB* and *thrC* increase from one copy number to four copies in the genome. Comparison of partial genes of strains TH and MG1655 are listed in Supplementary Table S4. The expression level of *thrAB* and *thrC* in L-threonine production was balanced by transforming the above plasmids into strain TH to generate strains 2929, 2935, 2901, 0129, 0101, 0135, 3529, 3535 and 3501. P_*flic*_, P_*1.1*_ and P_*2.1*_ promoter sequences were derived from reports (Jaishankar and Srivastava, 2020; Zhang et al., 2021). The *ptsG* gene deletion experiment was carried out by homologous recombination (Datsenko and Wanner, 2000). All fragments were amplified with Phanta HS ultra-fidelity DNA polymerase (Vazyme Biotech, Nanjing, China).

### RBS characterization

RBS characterization was achieved by monitoring the RFP fluorescence and cell density in real-time using a multiple detection microplate analyzer (SynergyHT, BioTek, Winooski, VT, United States). The details are as follows. The seeds of RBS characterization strains were prepared by transferring a single colony to a 12-well microassay plate containing 2 mL LB medium with 34 μg/mL chloramphenicol. The cells were grown at 37°C for 12 h. Next, 2% (v/v) of the seeds were inoculated into 0.2 mL LB medium with 34 μg/mL chloramphenicol in a 96-well microassay plate to detect red fluorescence. The 96-well plate was incubated at 37°C with oscillation. During RBS characterization, strain growth was measured at 600 nm. The red fluorescence was detected by excitation at 590 nm and emission at 645 nm.

### Medium and L-threonine fermentation

LB medium was used for plasmid construction and RBS strength characterization. L-threonine fermentation medium consists of 15 g/L (NH_4_)_2_SO_4_, 2 g/L KH_2_PO_4_, 1 g/L MgSO_4_·7H_2_O, 2 g/L yeast extract and 0.02 g/L FeSO4 (Kruse et al., 2002). Glucose (40 g/L) was added as the initial carbon source and 20 g/L CaCO_3_ was used to adjust the pH during fermentation. For shake flask fermentation, a single colony was incubated in fresh LB medium at 37°C for 12 h. The precultured seeds were then transferred with 1% (v/v) inoculation to a 300 mL shake flask that contained 20 mL fermentation medium. Fermentation was carried out at 220 rpm and 37°C. Cultures were supplemented with 40 g/L glucose when the glucose level was lower than 15 g/L.

The fed-batch culture was carried out in a 5-L bioreactor containing 4L medium (20 g/L (NH_4_)_2_SO_4_, 3 g/L yeast extract, 2 g/L KH_2_PO_4_, 2 g/L MgSO_4_·7H_2_O, 5 mg/L FeSO_4_·7H_2_O, 5 mg/L MnSO_4_·4H_2_O, 0.5 g/L betaine). Temperature was maintained at 37°C, the aeration rate at 1.5 vvm, pH was maintained automatically at 7.0 with NH_4_OH, and the dissolved oxygen value was maintained below 30%. 20 g/L initial amount of sterilized glucose was added in the working culture, and glucose concentration was controlled by continuous feeding.

### Analytical methods

One milliliter of the culture was mixed vigorously, and 0.1 mL was transferred to a 1.5 mL centrifuge tube. One millimolar HCl (0.9 mL) was added to this culture, and the sample was mixed to remove residual CaCO_3_. Subsequently, the OD_600_ was measured using a spectrophotometer (Shimadzu, Kyoto, Japan). For glucose and acetate assays, the cultures were centrifuged at 12,000 rpm for 2 min to collect the supernatant. The collected supernatant was filtered through a 0.22 μm water membrane for analysis. A refractive index detector (RID-10A; Shimadzu, Kyoto, Japan) and an AminexHPX-87H ion exclusion column (Bio-Rad Laboratories, Hercules, CA, United States) were used with 5 mM H_2_SO_4_ as the mobile phase and a flow rate of 0.6 mL/min.

For the detection of L-threonine, the collected supernatant was deproteinized with 5% trichloroacetic acid. Subsequently, the pretreated supernatant was derivatized with triethylamine and phenyl isothiocyanate, followed by extraction with *n*-hexane (Wang et al., 2022a). Briefly, 0.2 mL of sample and standard L-threonine were pretreated with a mixture of triethylamine-acetonitrile (1.4 mL of triethylamine mixed with 8.6 mL of acetonitrile). Next, we added phenylisothiocyanate-acetonitrile (25 μL of phenylisothiocyanate mixed with 2 mL of acetonitrile) to pretreat samples and the L-threonine standard for 1 h at room temperature. *n*-Hexane (0.4 mL) was added, and the sample was shaken vigorously. The lower layer (0.2 mL) was collected and diluted with 0.8 mL deionized water. The solution was filtered with a 0.22 μm organic membrane, and samples were detected using an HPLC equipped with a diode array detector (SPD-M20A; Shimadzu, Kyoto, Japan) and a VenusilAA (4.6 × 250 mm, 5 μm, AgelaTechanology) column at 40°C. The mobile phase consisted of (A) 15.2 g sodium acetate dissolved in 1850 mL of ultrapure water and mixed with 140 mL of acetonitrile and (B) 80% (v/v) acetonitrile and 20% (v/v) ultrapure water. The flow rate was 1 mL/min. The L-threonine concentration was quantified using the corresponding standard curve and peak area.

## Results and discussion

### Balancing the expression of *thrAB* and *thrC* to promote cell growth and L-threonine production

**FIGURE 2:**
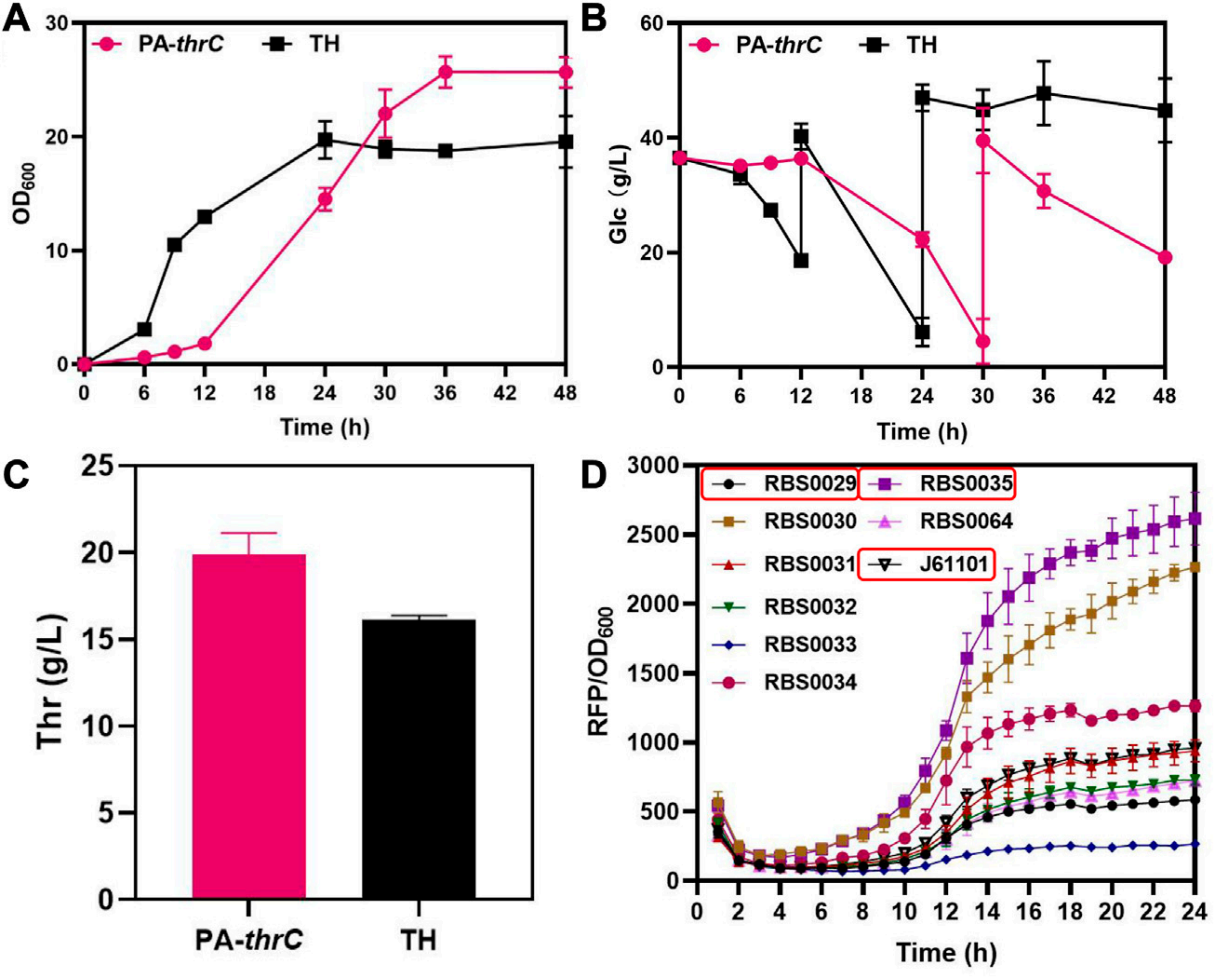
Comparison of the fermentation results of overexpressed *thrC* by medium copy PACYC-Duet plasmid and control TH and characterization results of different RBS intensities. The PA-ThrC strain overexpresses *thrC* on the PACYC-Duet plasmid. All results were derived from three (*n* = 3) independent repeats. **(A)** Comparison of cell growth (OD_600_) between overexpressing *thrC* and control strains. **(B)** Glucose consumption by the overexpressing *thrC* and control strains. **(C)** Comparison of L-threonine titer between the overexpressing *thrC* strain and the control strain after fermentation for 48 h. **(D)** The fluorescence intensity of RFP was monitored in real-time by a multi-detection microplate reader to characterize and screen RBS with different intensities. The RBS intensity was calculated using the ratio of RFP to OD_600_.

In the biosynthesis of L-threonine in *E. coli*, genes *thrA*, *thrB* and *thrC* encoding the last three key enzymes of this biosynthesis are located on the *thrABC* operon. ThrC catalyzes the final step of L-threonine synthesis, and this reaction is slow (Lee et al., 2013). As the rate-limiting step of L-threonine production, it is necessary to enhanced the expression of *thrC*. We used the L-threonine-producing strain TH available from FuFeng Group as the initial strain, which was derived from MG1655 (Supplementary Table S2). Initially, we enhanced the expression of *thrC* on the low copy PCL-1920 plasmid and transformed the plasmid into the initial strain TH to obtain the pcl-*thrC* strain. However, the production of L-threonine and cell growth of the strain was not significantly improved compared with the original strain TH (Supplementary Figure S1A,B). We analyzed that the reason is the weak degree of enhanced expression. Next, we enhanced the expression of *thrC* on the medium copy PACYC-Duet plasmid and transformed the plasmid into the initial strain TH to obtain the PA-*thrC* strain. Although the production of L-threonine increased, cell growth and the glucose consumption rate were seriously impeded during early cultivation (Figures 2A–C). In addition, *thrA*, *thrB*, *thrC* are located on the *thrABC* operon. So the overexpressed *thrC* might disrupt the balance between *thrAB* and *thrC*. Therefore, we analyzed the influence of various ratios of *thrAB* and *thrC* on L-threonine synthesis and cell growth. Ribosome binding sites (RBS) define the translation efficiency of genes and are typically used in gene regulation studies (Hur et al., 2020; Wang et al., 2022b). We selected three RBS with different intensities by fluorescence characterization, RBS0035 (2615), J61101 (958) and RBS0029 (585), to regulate *thrAB* and *thrC* expression levels, with an intensity ratio between them of 13:5:3 (Figure 2D). We constructed nine strains containing various combinations and levels of *thrAB* and *thrC* expression.

**FIGURE 3:**
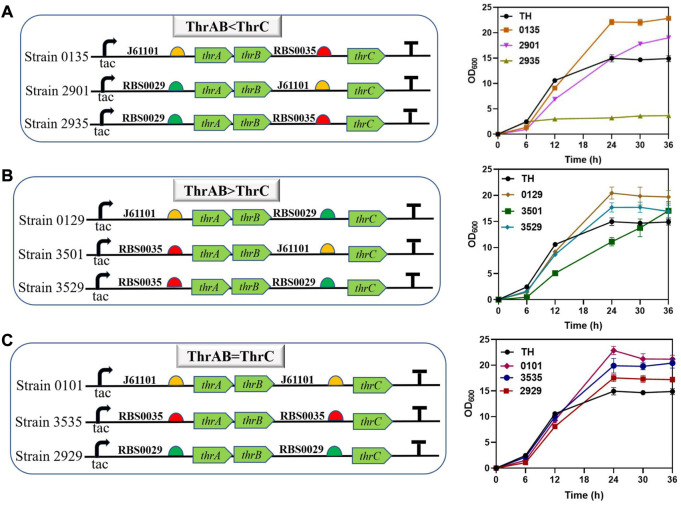
The effect of different expression intensities of *thrAB* and *thrC* on strain TH growth. According to the RBS strength, the adjusted expression of *thrAB* and *thrC* was classified into three groups: ThrAB < ThrC, ThrAB > ThrC and ThrAB = ThrC. All results were calculated from three (*n* = 3) independent repeats. **(A)** Strains with *thrAB* expression weaker than *thrC* were constructed using different combinations of RBS, and fermentation results OD_600_ are shown. **(B)** Strains with *thrAB* expression stronger than *thrC* were constructed by using different combinations of RBS, and fermentation results OD_600_ are shown. **(C)** Strains with equal expression strengths of *thrAB* and *thrC* were constructed by using different combinations of RBS, and fermentation results OD_600_ are shown.

**FIGURE 4:**
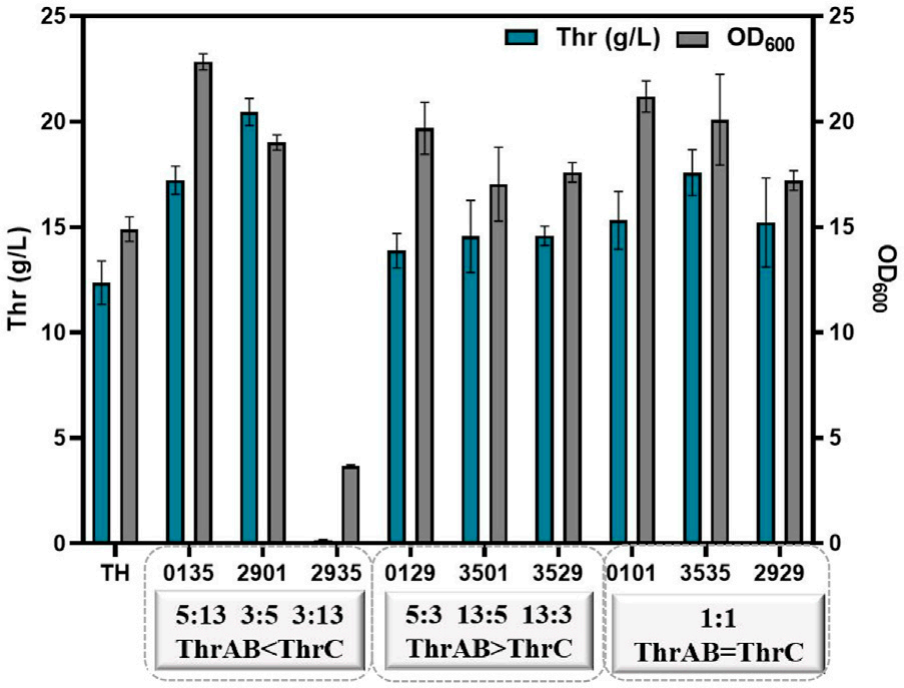
The effect of different expression intensities of *thrAB* and *thrC* on L-threonine production by the TH strain. All results were derived from three (*n* = 3) independent repeats.

The results showed that different combinations of *thrAB* and *thrC* expression levels had different effects on cell growth. High expression of *thrC* promoted growth and L-threonine accumulation simultaneously; however, there were also ratios between *thrAB* and *thrC* when the level of L-threonine decreased because the proportion of *thrC* was too high. Among the nine strains constructed, the 2901 strain (*thrAB*:*thrC* = 3:5) yielded the highest L-threonine titer. The 2935 strain (3:13) grew slowly because of the large difference in the expression levels of *thrAB* and *thrC*. Optimal growth was observed for the 0135 strain (5:13) (Figure 3A; Figure 4). Accumulation of L-threonine was poor when *thrC* was expressed weakly, and different ratios had minimal effect on L-threonine production (Figure 3B, Figure 4). Equal expression levels of *thrAB* and *thrC* in strain 3535 yielded a relatively good L-threonine titer, but production was lower than that of strain 2901 (Figure 3C, Figure 4). The results indicated that high expression of *thrC* in an appropriate *thrAB*:*thrC* ratio yielded optimal cell growth and L-threonine production.

In summary, the results showed that when the expression of *thrC* was higher than *thrAB*, it was beneficial to the growth and production of L-threonine, and the level of L-threonine decreased when the expression of *thrC* was much higher than *thrAB* in particular strains. Using a *thrAB*:*thrC* expression ratio of 3:5 yielded the highest production of L-threonine. Production of L-threonine by proportional expression of *thrAB* and *thrC* was found to be a more effective approach to produce L-threonine than direct overexpression of *thrABC* (Zhao et al., 2018).

### Dynamic regulation of the engineered *thrABC* to counter metabolic burden and increase L-threonine productivity

As described above, L-threonine production was promoted by carefully regulating the expression levels of *thrAB* and *thrC* and optimizing the *thrAB*:*thrC* ratio; however, constitutive overexpression of the *thrABC* operon inhibited cell growth (Figures 3A–C). We adopted a dynamic regulation strategy to ensure that the metabolic burden caused by the premature introduction of the *thrABC* operon was alleviated. Instead of using IPTG and other chemical inducers, a growth-related promoter was used to control *thrABC* expression after obtaining a high cell density. Several stationary phase promoters with different strengths have been obtained previously by random mutagenesis of a wild-type stationary phase promoter (Shimada et al., 2004). The strength of the P_*1.1*_ and P_*2.1*_ promoters obtained was equivalent to *E. coli* promoters (Jaishankar and Srivastava, 2020). The promoters of *fliA*, *fliC* and *flgC* involved in flagella construction were also identified as stationary phase promoters. The transcriptional intensity of these promoters is active during the lag and early exponential phases but inhibited throughout the late exponential and stationary phases (Zhang et al., 2019).

**FIGURE 5:**
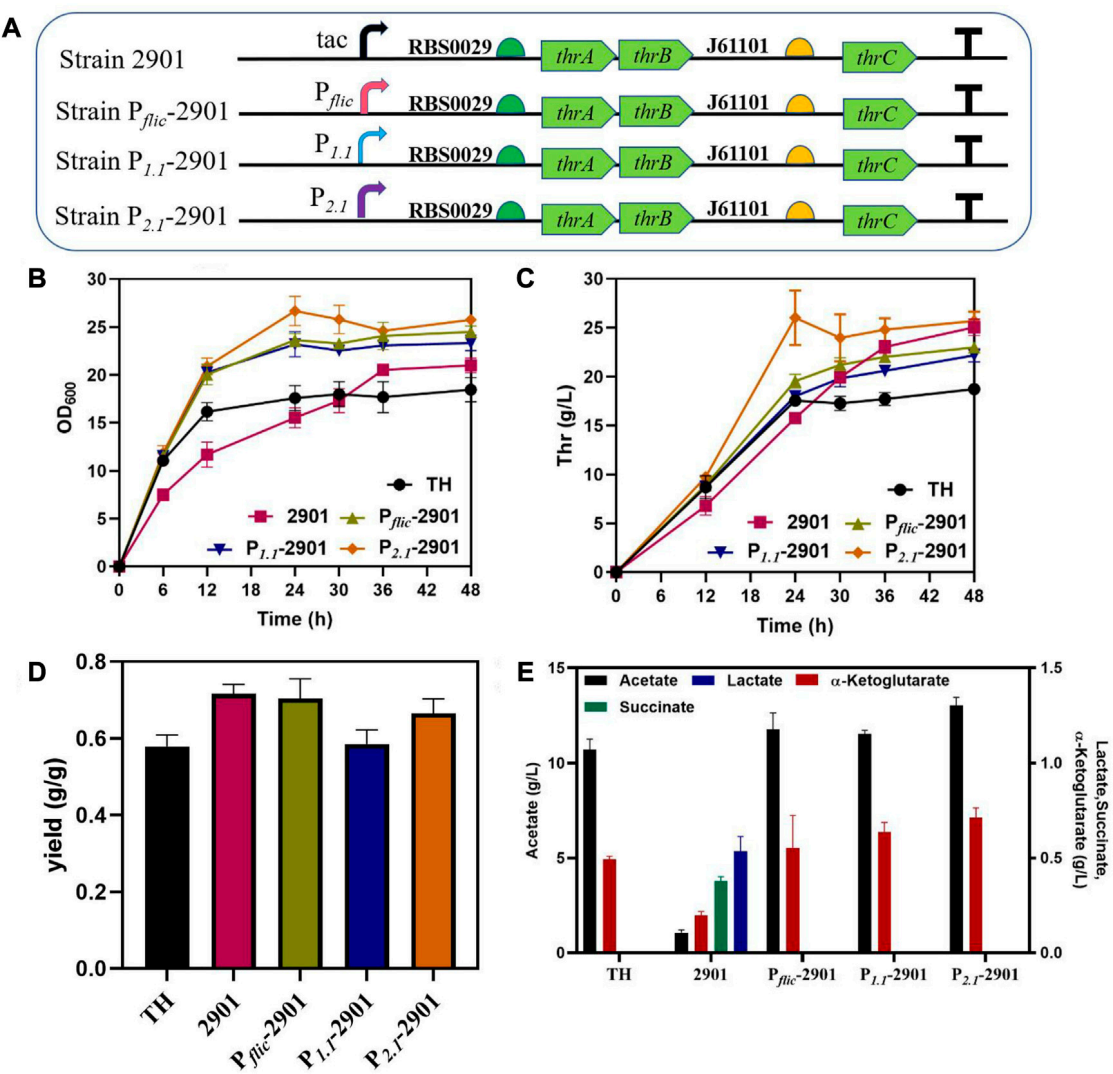
The effect of dynamic control of the *thrABC* operon with a stationary phase promoter on the growth and L-threonine production in *Escherichia coli* TH. All results were calculated from three (*n* = 3) independent repeats. **(A)** The strain was constructed by replacing the tac promoter with different promoters. **(B)** The OD_600_ of fermenting strains is controlled by a stationary phase promoter. **(C)** Samples were taken at 12, 24, 30, 36 and 48 h during fermentation to measure L-threonine production. **(D)** The yield of the final product was calculated by the ratio of glucose consumption to L-threonine titer. **(E)** The content of the by-product acetate and other metabolites at the end of fermentation for 48 h.

The effect of dynamic regulation of *thrABC* on strain growth and production was verified by using three stationary phase promoters, P_*fliC*_, P_*1.1*_ and P_*2.1*_, for *thrABC* regulation in the PA-29*thrAB-*01*thrC* plasmid (Figure 5A). The results showed that the strains controlled by the three stationary phase promoters displayed improved growth in the early stage, and L-threonine production increased significantly compared with the control group. Among these strains, growth was strongest for strain P_*2.1*_-2901, which was controlled by P_*2.1*_, and L-threonine production was similar to that of the strain controlled by the tac promoter (Figures 5B,C). This observation is because the P_*2.1*_ promoter is the strongest among the three tested (Jaishankar and Srivastava, 2020). We measured L-threonine production in each period and found that L-threonine accumulation in the P_*2.1*_-2901 strain was the fastest, with the L-threonine titer and productivity reaching 26.02 g/L and 1.08 g/L/h at 24 h, respectively. L-threonine production by strain P_*2.1*_-2901 was 44.56% and 65.00% higher than that of the control strain TH and strain 2901 controlled by the tac promoter, respectively (Figure 5C). This result arises because the replaced stationary phase promoter is more active during the early exponential phase, which promotes the overexpression of the *thrABC* operon to accelerate the accumulation of L-threonine. This phenomenon suggests that this strategy can enhance product titer and shorten the fermentation period. The overexpression of the *thrABC* gene controlled by the stationary phase promoter can improve L-threonine yield (Figure 5D).

Although we increased L-threonine productivity by using a stationary phase promoter, the strains with better growth in the early stage also produced a large amount of acetate (Figure 5E). Studies have suggested that the rapid use of glucose during the early stage of cell growth will lead to an acetate overflow (Eiteman and Altman, 2006). Thus, we next tried to reduce the acetate overflow.

### Decreasing glucose transport to reduce acetate overflow and improve L-threonine production

The phosphotransferase system (PTS) is the major glucose transport system in *E. coli*. Deletion of PTS has been reported to reduce acetate overflow and increase the production of recombinant proteins and biochemicals (Wang et al., 2006; Kang et al., 2009). We deleted the *ptsG* gene in three threonine-producing strains, TH, 2901 and P_*2.1*_-2901, to examine the effect of PTS activity on L-threonine production.

**FIGURE 6:**
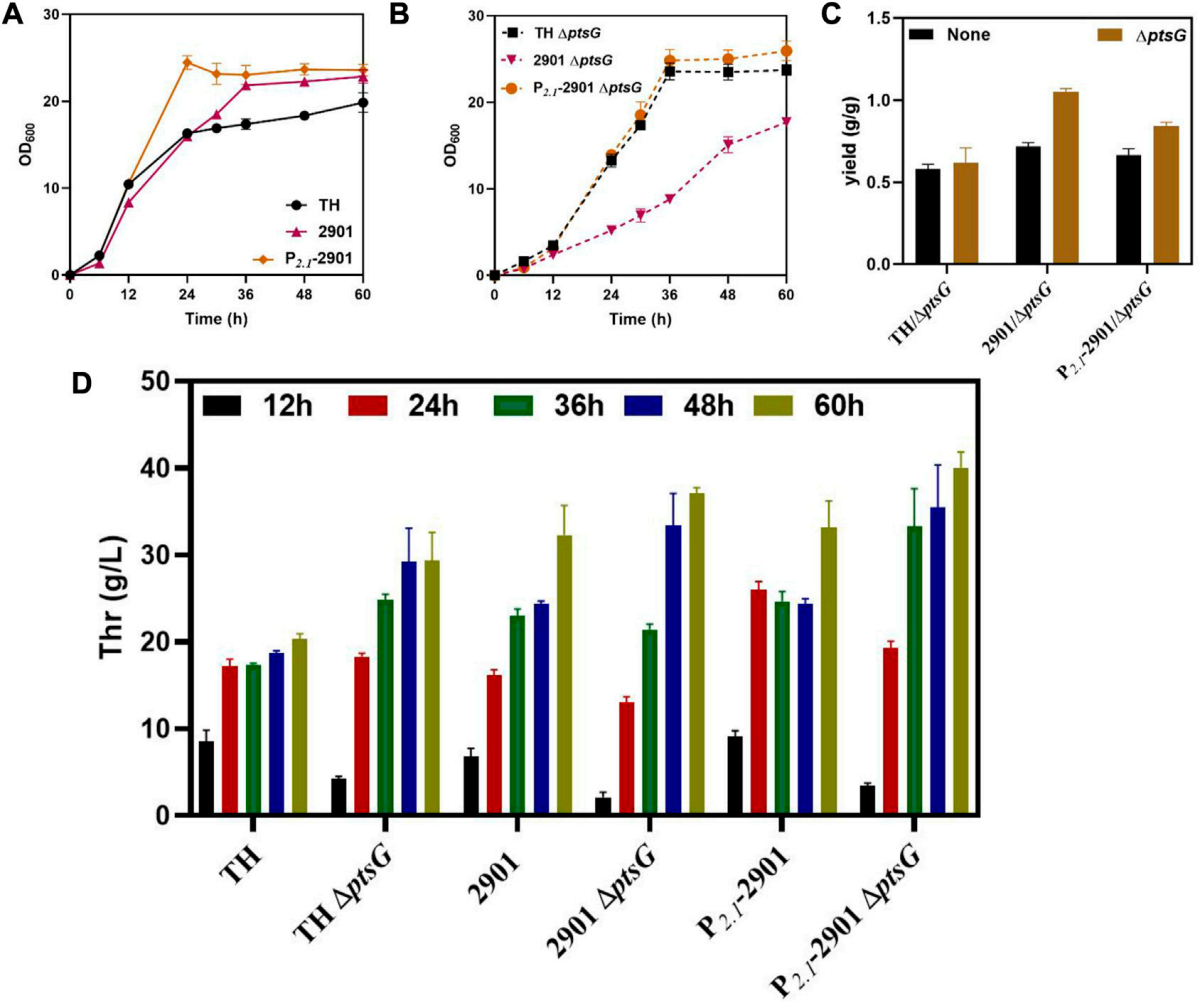
The effect of deleting *ptsG* on the growth and L-threonine production of strain TH. All results were determined from three (*n* = 3) independent repeats. **(A)** Growth of strains before *ptsG* gene deletion. **(B)** Growth of strains after *ptsG* gene deletion. **(C)** The yield of the final product was calculated by the glucose consumption and L-threonine titer. **(D)** During fermentation, samples taken at 12, 24, 36, 48 and 60 h were used to measure L-threonine production.

The growth of the three strains was retarded after deleting *ptsG*, which was caused by the decrease in the glucose uptake rate by the cells. The growth of the 2901Δ*ptsG* strain was inhibited significantly because of the combined effects of *thrABC* overexpression and *ptsG* deletion. Optimal growth was observed for strain P_*2.1*_-2901*ΔptsG*, reaching a stable growth state after 36 h, whereas the 2901Δ*ptsG* strain showed slower growth up to 60 h (Figures 6A,B). This observation also reflects the advantages of dynamic regulation. Using the P_*2.1*_-2901Δ*ptsG* strain can shorten the fermentation period and increase L-threonine production. L-threonine production by the three strains was improved when compared with the corresponding strains without the *ptsG* deletion, with an increase in L-threonine yield (Figure 6C). At 60 h, L-threonine production by strain P_*2.1*_-2901Δ*ptsG* reached 40.06 g/L, which was 36.30% and 7.95% higher than that of THΔ*ptsG* and 2901Δ*ptsG* strains, respectively (Figure 6D). As expected, acetate was not generated during fermentation.

**FIGURE 7:**
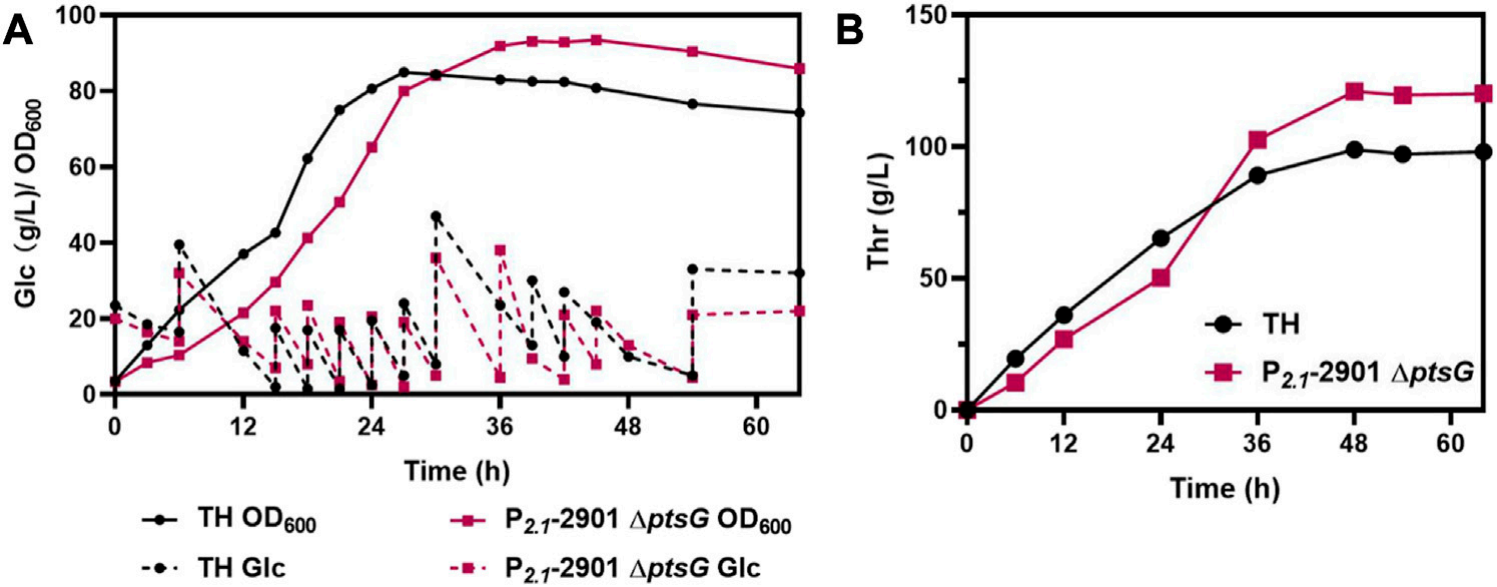
The fed-batch process with P_*2.1*_-2901Δ*ptsG* and TH in a 5-L bioreactor. Biomass, glucose consumption, and L-threonine titer were monitored in real time. **(A)** Biomass and glucose consumption of strains. **(B)** L-threonine titer of strains.

To further verify the properties of P_*2.1*_-2901Δ*ptsG* for L-threonine production, fed-batch fermentation was performed in 5-L bioreactor, using TH as a control. The cell density, glucose consumption levels (Figure 7A) and L-threonine titer (Figure 7B) are depicted. Following 48 h of cultivation, P_*2.1*_-2901Δ*ptsG* could produce 121.05 g/L L-threonine, leading to a yield of 0.60 g/g glucose and productivity of 2.52 g/L/h. TH could produce 98.88 g/L L-threonine in 48 h, with a yield and productivity of 0.49 g/g glucose and 2.06 g/L/h, respectively. After 48 h of incubation, unfavorable growth conditions and irreparable cellular damage lead to a death phase and long-term stationary phase (Finkel, 2006), resulting in the lose viability of L-threonine production and glucose consumption. In contrast to the results of other reports, P_*2.1*_-2901Δ*ptsG* achieved the highest productivity and yield. Therefore, with respect to the three major parameters in industrial production, P_*2.1*_-2901Δ*ptsG* possesses substantial L-threonine production ability.

The reaction catalyzed by ThrC is the rate-limiting step of L-threonine production and overexpression of *thrC* inhibits cell growth. Here, we showed that balanced regulation of *thrAB* and *thrC* expression levels promotes *E. coli* growth and L-threonine production. L-threonine production was further improved by dynamically regulating the overexpression of *thrABC*. Finally, the deletion of *ptsG* reduced acetate production and further increased the titer and yield of L-threonine. With the above approaches, we obtained strain P_*2.1*_-2901Δ*ptsG*, with the titer of L-threonine by fermentation in a shake flask for 60 h reaching 40.06 g/L, which is 96.85% higher than that of the control strain TH (20.35 g/L) and the highest titer reported in a shake flask (Table 1). The yield reached 0.84 g/g, which was 44.82% higher than the control strain TH (0.58 g/g). P_*2.1*_-2901Δ*ptsG* could produce 121.05 g/L L-threonine in 48 h, with a yield and productivity of 0.60 g/g glucose and 2.52 g/L/h by fed-batch fermentation, respectively. The maximum L-threonine yield and productivity was obtained in reported fed-batch fermentation, and L-threonine titer is close to the maximum (127.30 g/L) (Table 1). This study provided a new concept for improving L-threonine production and downstream products.

**TABLE 1:** L-threonine production using different strategies.

Species	Shake flasks fermentation			Fed-batch fermentation			Source
	Titer (g/L)	Yield (g/g)	Productivity (g/L/h)	Titer (g/L)	Yield (g/g)	Productivity (g/L/h)	
*E. coli* (TWF083)	29.73	-	-	116.62	0.49	2.43	Zhao et al. (2020)
*E. coli* (TWF044)	28.49	0.72	-	103.89	0.45	-	Yang et al. (2019)
*E. coli* (TSW009)	26.00	0.65	0.54	-	-	-	Wang et al. (2022b)
*E. coli* (TWF113 / pFT24rpa 1)	25.85	-	-	-	-	-	Fang et al. (2020)
*E. coli* (JLTHR)	-	-	-	127.30	0.58	-	Su et al. (2018)
*E. coli* (WMZ016/pFW01-thrA*BC-rhtC)	17.98	0.35	-	-	-	-	Zhu et al. (2019)
*Halomonas bluephagenesis (TDHR3-42-p226)*	7.50	-	-	33.00		1.40	Du et al. (2020)
*Cotynebacterium glutamicum*	12.80	-	-		-	-	Wei et al. (2018)
*E. coli (P*_2.1_*-2901ΔptsG)*	40.06	0.84	0.67	121.05	0.60	2.52	This Study

## Data Availability

The original contributions presented in the study are included in the article/Supplementary Material, further inquiries can be directed to the corresponding author.
