# Supplementary material for: Dynamic and balanced regulation of the thrABC operon gene for efficient synthesis of L-threonine
Source: Front Bioeng Biotechnol. 2023 Mar 2;11:1118948. doi: 10.3389/fbioe.2023.1118948 (PMC10018013; doi:10.3389/fbioe.2023.1118948)
Supplement: Supplementary file 1 [file DataSheet1.docx]

**Supplementary Material**

**Supplementary Table S1.** Plasmids used in this study

| **Plasmids** | **Description** | **Source** |
| --- | --- | --- |
| PACYC-Duet | rep_p15A_ Cm^R^ P_lac_ | Lab Stock |
| PA-*RFP*-0029 | rep_p15A_ Cm^R^ P*_tac_*B0029‐*rfp* | This study |
| PA-*RFP*-0030 | rep_p15A_ Cm^R^ P*_tac_*B0030‐*rfp* | This study |
| PA-*RFP*-0031 | rep_p15A_ Cm^R^ P*_tac_*B0031‐*rfp* | This study |
| PA-*RFP*-0032 | rep_p15A_ Cm^R^ P*_tac_*B0032‐*rfp* | This study |
| PA-*RFP*-0033 | rep_p15A_ Cm^R^ P*_tac_*B0033‐*rfp* | This study |
| PA-*RFP*-0034 | rep_p15A_ Cm^R^ P*_tac_*B0034‐*rfp* | This study |
| PA-*RFP*-0035 | rep_p15A_ Cm^R^ P*_tac_*B0035‐*rfp* | This study |
| PA-*RFP*-0064 | rep_p15A_ Cm^R^ P*_tac_*B0064‐*rfp* | This study |
| PA-*RFP*-J61101 | rep_p15A_ Cm^R^ P*_tac_*J61101‐*rfp* | This study |
| PA-29*thrAB-*29*thrC* | rep_p15A_ Cm^R^ P*_tac_*B0029-*thrAB-*B0029-*thrC* | This study |
| PA-29*thrAB-*01*thrC* | rep_p15A_ Cm^R^ P*_tac_*B0029-*thrAB-*J61101-*thrC* | This study |
| PA-29*thrAB-*35*thrC* | rep_p15A_ Cm^R^ P*_tac_*B0029-*thrAB-*B0035-*thrC* | This study |
| PA-01*thrAB-*29*thrC* | rep_p15A_ Cm^R^ P*_tac_*J61101-*thrAB-* B0029-*thrC* | This study |
| PA-01*thrAB-*01*thrC* | rep_p15A_ Cm^R^ P*_tac_*J61101-*thrAB-*J61101-*thrC* | This study |
| PA-01*thrAB-*35*thrC* | rep_p15A_ Cm^R^ P*_tac_*J61101-*thrAB-*B0035-*thrC* | This study |
| PA-35*thrAB-*2 *thrC* | rep_p15A_ Cm^R^ P*_tac_*B0035-*thrAB-* B0029-*thrC* | This study |
| PA-35*thrAB-*01*thrC* | rep_p15A_ Cm^R^ P*_tac_*B0035-*thrAB-*J61101-*thrC* | This study |
| PA-35*thrAB-*35*thrC* | rep_p15A_ Cm^R^ P*_tac_*B0035-*thrAB-* B0035-*thrC* | This study |
| PA-P*_flic_*-29AB-01C | rep_p15A_ Cm^R^ P*_flic_*B0029-*thrAB-*J61101-*thrC* | This study |
| PA-P*_1.1_*-29AB-01C | rep_p15A_ Cm^R^ P*_1.1_*B0029-*thrAB-*J61101-*thrC* | This study |
| PA-P*_2.1_*-29AB-01C | rep_p15A_ Cm^R^ P*_2.1_*B0029-*thrAB-*J61101-*thrC* | This study |
| PTKred | rep_pSC101_ Spc^R^ lac-inducible expression; DNA repair protein from *E. coli*, RecA | Lab Stock |
| PCP20 | rep_pSC101_ Cm^R^ Amp^R^ site-specific recombinase, FLP; temperature-sensitive variant of the phage λ repressor | Lab Stock |

**Supplementary Table S2.** Strains used in this study

| **Strains** | **Description** | **Source** |
| --- | --- | --- |
| *E. coli* DH5α | F‐ supE44 ΔlacU169 (ϕ80 lacZΔM15) hsdR17 recA1 endA1 gyrA96 thi1 relA1 | Invitrogen |
| *E. coli* K-12 MG1655  TH | K12 F‐ lambda‐ ilvG‐ rfb‐50 rph‐1  L‐threonine production host cell | Invitrogen  FuFeng Group |
| 2929 | TH carrying PA-29*thrAB-*29*thrC* | This study |
| 2901 | TH carrying PA-29*thrAB-*01*thrC* | This study |
| 2935 | TH carrying PA-29*thrAB-*35*thrC* | This study |
| 0129 | TH carrying PA-01*thrAB-*29*thrC* | This study |
| 0101 | TH carrying PA-01*thrAB-*01*thrC* | This study |
| 0135 | TH carrying PA-01*thrAB-*35*thrC* | This study |
| 3529 | TH carrying PA-35*thrAB-*29*thrC* | This study |
| 3501 | TH carrying PA-35*thrAB-*01*thrC* | This study |
| 3535 | TH carrying PA-35*thrAB-*35*thrC* | This study |
| P*_flic_*-2901 | TH carrying PA-P*_flic_*-29AB-01C | This study |
| P*_1.1_*-2901 | TH carrying PA-P*_1.1_*-29AB-01C | This study |
| P*_2.1_*-2901 | TH carrying PA-P*_2.1_*-29AB-01C | This study |
| Duet*ΔptsG* | TH*ΔptsG* carrying PACYC-Duet | This study |
| 2901*ΔptsG* | TH*ΔptsG* carrying PA-29*thrAB-*01*thrC* | This study |
| P*_flic_*-2901*ΔptsG* | TH*ΔptsG* carrying PA-P*_flic_*-29AB-01C | This study |
| P*_1.1_*-2901*Δ*ptsG | TH*ΔptsG* carrying PA-P*_1.1_*-29AB-01C | This study |
| P*_2.1_*-2901*Δ*ptsG | TH*ΔptsG* carrying PA-P*_2.1_*-29AB-01C | This study |

**Supplementary Table S3.** Primers used in this study

| **Primer name** | **Sequences(5’‐3’)** |
| --- | --- |
| 34ThrA-6.14F | TAATGTCTAGAGAAAGAGGAGAAATACTAGATGCGAGTGTTGAAGTTCGG |
| PA-AA-6.2F | CTCGAGTCTGGTAAAGAAACCGC |
| CE-mCherry-6.5F | TCTGATAAGAGACACCGGCATACT |
| CE-mCherry-6.5R | TGCTGGTTTACCGGTTTATTGACTAC |
| thrC-6.14R | CAGCAGCGGTTTCTTTACCAGACTCGAGTTACTGATGATTCATCATCAATTTACGCAAC |
| 29ThrA-6.14 | TAATGTCTAGAGTTCACACAGGAAACCTACTAGATGCGAGTGTTGAAGTTCGG |
| 29AA-6.2R | CTAGTAGGTTTCCTGTGTGAACTCTAGACATTATACGAGCCGATGATTAATTGTCAAA |
| 01ThrC-6.14F | TAATCTAGAGAAAGACAGGACCCACTAGTATGAAACTCTACAATCTGAAAGATCACAAC |
| 01ThrB-6.14R  29ThrC-6.14F  29thrAB-6.14R  34ThrC-6.14F  34ThrB-6.14R  35ThrC-6.14F  35ThrB-6.14R  34ThrA-6.14F  34AA-6.2R  35ThrA-6.14F  35AA-6.2R  01ThrA-6.14F  01AA-6.2R  29mCherry-6.2F  30mCherry-6.2F  30AA-6.2R  31mCherry-6.2F  31AA-6.2R  32mCherry-6.2F  32AA-6.2R  33mCherry-6.2F  33AA-6.2R  34mCherry-6.2F  34AA-6.2R  35mCherry-6.2F  35AA-6.2R  64mChery-6.2F  64AA-6.2R  01mCherry-6.2F  01AA-6.2R  AA1.1-10.19R  1.1-10.19F  P1.1-10.19R  AA2.1-10.19R  2.1-10.19F  P2.1-10.19R  mCherry-6.2R  PfliC-10.19F  AA-10.19R  fliC-10.19F  PfliC-10.19R  upptsG-8.26F  CE-ptsG-10.5F  pKD3-F  upptsG-8.26R  downptsG-8.26F  pKD3-R  downptsG-8.26R  CE-ptsG9.20R | TCATACTAGTGGGTCCTGTCTTTCTCTAGATTAGTTTTCCAGTACTCGTGCGC  ATCTAGAGTTCACACAGGAAACCTACTAGATGAAACTCTACAATCTGAAAGATCACAAC  ATCTAGTAGGTTTCCTGTGTGAACTCTAGATTAGTTTTCCAGTACTCGTGCGC  CTAATCTAGAGAAAGAGGAGAAATACTAGATGAAACTCTACAATCTGAAAGATCACAAC  TTCATCTAGTATTTCTCCTCTTTCTCTAGATTAGTTTTCCAGTACTCGTGCGC  AATCTAGAGATTAAAGAGGAGAATACTAGATGAAACTCTACAATCTGAAAGATCACAAC  CATCTAGTATTCTCCTCTTTAATCTCTAGATTAGTTTTCCAGTACTCGTGCGC  TAATGTCTAGAGAAAGAGGAGAAATACTAGATGCGAGTGTTGAAGTTCGG  CTAGTATTTCTCCTCTTTCTCTAGACATTATACGAGCCGATGATTAATTGTCAAAT  TAATGTCTAGAGATTAAAGAGGAGAATACTAGATGCGAGTGTTGAAGTTCGG  CTAGTATTCTCCTCTTTAATCTCTAGACATTATACGAGCCGATGATTAATTGTCAAA  TAATGTCTAGAGAAAGACAGGACCCACTAGTATGCGAGTGTTGAAGTTCGG  ACTAGTGGGTCCTGTCTTTCTCTAGACATTATACGAGCCGATGATTAATTGTCAAA  TCTAGAGTTCACACAGGAAACCTACTAGATGGTGAGCAAGGGCGAG  TGTCTAGAGATTAAAGAGGAGAAATACTAGATGGTGAGCAAGGGCGAG  CTAGTATTTCTCCTCTTTAATCTCTAGACATTATACGAGCCGATGATTAATTGTCAA  TGTCTAGAGTCACACAGGAAACCTACTAGATGGTGAGCAAGGGCGAG  CTAGTAGGTTTCCTGTGTGACTCTAGACATTATACGAGCCGATGATTAATTGTCAA  ATGTCTAGAGTCACACAGGAAAGTACTAGATGGTGAGCAAGGGCGAG  CTAGTACTTTCCTGTGTGACTCTAGACATTATACGAGCCGATGATTAATTGTCAA  ATAATGTCTAGAGTCACACAGGACTACTAGATGGTGAGCAAGGGCGAG  CTAGTAGTCCTGTGTGACTCTAGACATTATACGAGCCGATGATTAATTGTCAAAT  AATGTCTAGAGAAAGAGGAGAAATACTAGATGGTGAGCAAGGGCGAG  CTAGTATTTCTCCTCTTTCTCTAGACATTATACGAGCCGATGATTAATTGTCAAAT  ATGTCTAGAGATTAAAGAGGAGAATACTAGATGGTGAGCAAGGGCGAG  CTAGTATTCTCCTCTTTAATCTCTAGACATTATACGAGCCGATGATTAATTGTCAAA  AATGTCTAGAGAAAGAGGGGAAATACTAGATGGTGAGCAAGGGCGAG  CTAGTATTTCCCCTCTTTCTCTAGACATTATACGAGCCGATGATTAATTGTCAAAT  ATGTCTAGAGAAAGACAGGACCCACTAGTATGGTGAGCAAGGGCGAG  ACTAGTGGGTCCTGTCTTTCTCTAGACATTATACGAGCCGATGATTAATTGTCAAA  CGACCGTCATTTCCTAATGCAGGAGTCGCATAAGGGAGAGCGTCGAGATCC  AAAGTCTATGATTGCGAAACAACAACGTTCTAGAGTTCACACAGGAAACCTACTAGATG  ACGTTGTTGTTTCGCAATCATAGACT  GCGACCGTCATTTCCTAATGCAGGAGTCGCATAAGGGAGAGCGTCGAGATCC  AAAGTCTATGATTGCGAAACAACAACGTTCTAGAGTTCACACAGGAAACCTACTAGATG  ACGTTGTTGTTTCGCAATCATAGACT  CGCAGCAGCGGTTTCTTTACCAGACTCGAGCTACTTGTACAGCTCGTCCATGCC  ATGCGACTCCTGCATTAGGAAAT  CCATTTTTTATTTCCTAATGCAGGAGTCGCATAAGGGAGAGCGTCGAGATCC  CAACGACTTGCAATATAGGATAACGAATCTCTAGAGTTCACACAGGAAACCTACTAGAT  GATTCGTTATCCTATATTGCAAGTCGTTG  ATTGGTCGTGAACTGAACAAGCC  CTTCTCCAATGATCTGAAGTTGAAACGT  GGCTGACATGGGAATTAGCCAT  TATGGACCATGGCTAATTCCCATGTCAGCCAATTGAGAGTGCTCCTGAGTATGGG  GCCTACACAATCGCTCAAGACGTGTAATGCTCCGTAAGACGTTGGGGAGACTAAG  GCATTACACGTCTTGAGCGATTG  GATCGTGACAGTTCAGGCAAATATCTG  TCAGTTCATGGGCCAACATCTTC |

**Supplementary Table S4.** Comparison of partial genes of strains TH and MG1655

|  | **TH** | **MG1655** |
| --- | --- | --- |
| ***asd*** | **4** | **1** |
| ***thrA*** | **4 (G1297A)** | **1** |
| ***thrB*** | **4** | **1** |
| ***thrC*** | **4** | **1** |
| ***pntAB*** | **2** | **1** |
| ***ppc*** | **2** | **1** |
| ***aspC*** | **7** | **1** |
| ***thrL*** | **0** | **1** |
| ***rhtABC*** | **1** | **1** |
| ***aspA*** | **2 (A699G)** | **1** |
| ***tdh*** | **0** | **1** |
| ***tdcC*** | **1** | **1** |


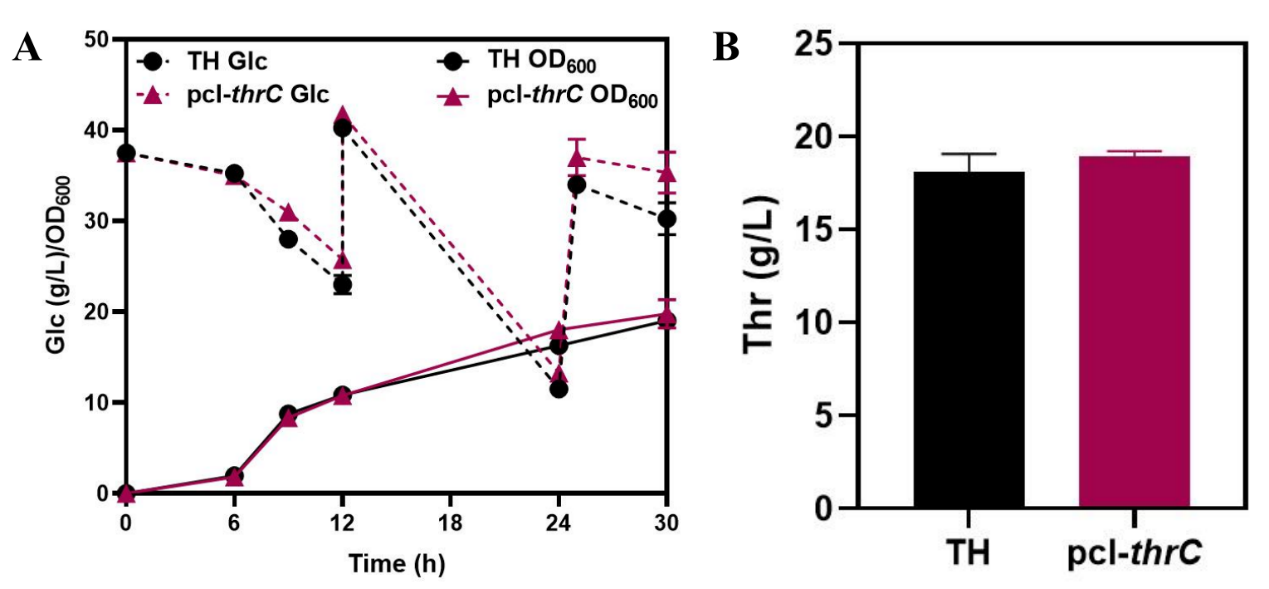
**Supplementary Figure S1.** Comparison of the fermentation results of overexpressed *thrC* by low copy PCL1920 plasmid and control TH. **(A)** Comparison of cell growth (OD_600_) and glucose consumption between overexpressing *thrC* and control strains. **(B)** Comparison of L-threonine titer between the overexpressing *thrC* strain and the control strain after fermentation for 30 h.
